# Neuro-ophthalmic sarcoidosis with extraocular-muscle involvement: neuroimaging

**DOI:** 10.1055/s-0046-1827163

**Published:** 2026-08-31

**Authors:** Lucas Yunes Cominatto Barbosa, Luiza Maretti Scomparin, Rafael Luccas, Fabiano Reis

**Affiliations:** 1 Universidade Estadual de Campinas, Faculdade de Ciências Médicas, Departamento de Radiologia e Oncologia, Campinas SP, Brazil.; 2 Universidade Estadual de Campinas, Faculdade de Ciências Médicas, Departamento de Oftalmologia e Otorrinolaringologia, Campinas SP, Brazil.

**Figure 1:**
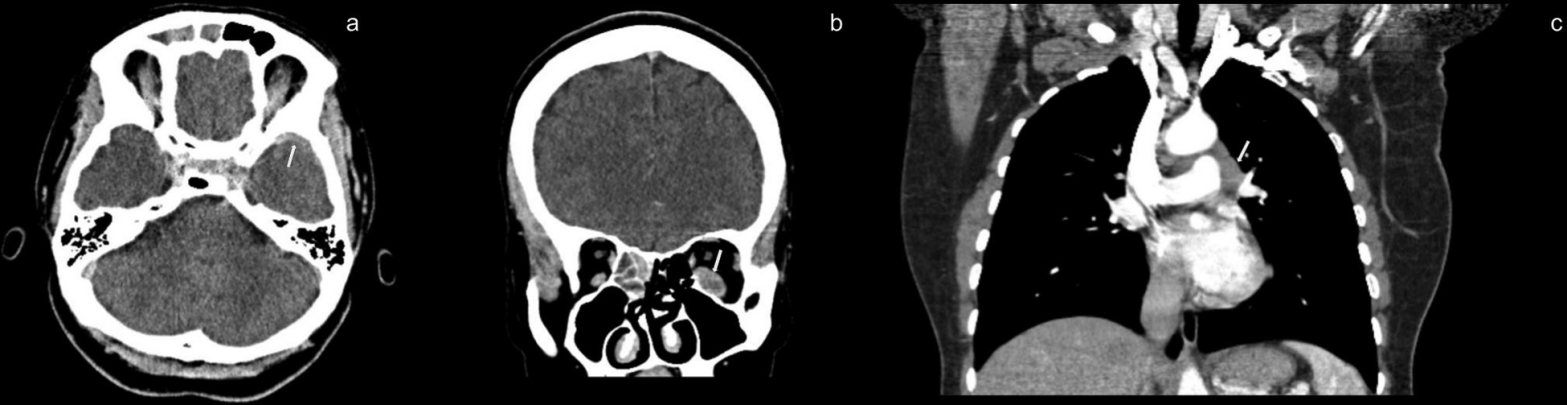
Axial contrast-enhanced head computed tomography (CT) scan showing focal pachymeningeal thickening and enhancement along the left temporal region (arrow in **A** ). Coronal contrast-enhanced head CT demonstrating enlargement of the left inferior rectus and inferior oblique muscles (arrow in **B** ). Coronal contrast-enhanced chest CT revealing left hilar and paratracheal lymphadenopathy (arrow in **C** ).

**Figure 3:**
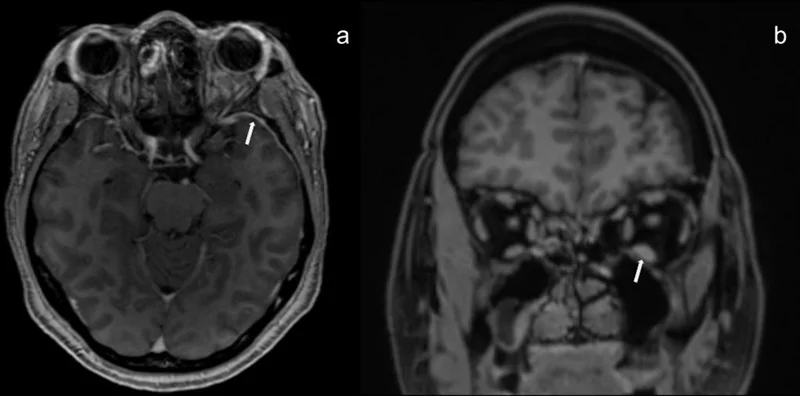
Follow-up brain MRI scan after treatment, demonstrating marked reduction of pachymeningeal (arrow in **A** ) and left inferior rectus and inferior oblique muscle thickening and enhancement (arrow in **B** ) on postcontrast T1-weighted imaging.

A 50-year-old woman presented with severe headache, diplopia, photophobia, and painful left ophthalmoplegia. The initial laboratory tests and cerebrospinal fluid analysis were unremarkable. 1 Neuroimaging revealed left temporal pachymeningeal thickening with homogeneous contrast enhancement and hypointensity on T2-weighted images, associated with enlargement of the left inferior rectus muscle. Chest computed tomography and positron-emission tomograpy–computed tomography scans demonstrated mediastinal and hilar lymphadenopathy with increased metabolic activity ( Figures 1 2 ). 2 The histopathological analysis of a mediastinal lymph node showed non-necrotizing granulomas, confirming sarcoidosis. The corticosteroid therapy resulted in rapid clinical improvement and near-complete resolution of the imaging abnormalities on a follow-up magnetic resonance imaging scan ( Figure 3 ). 3 4

**Figure 2:**
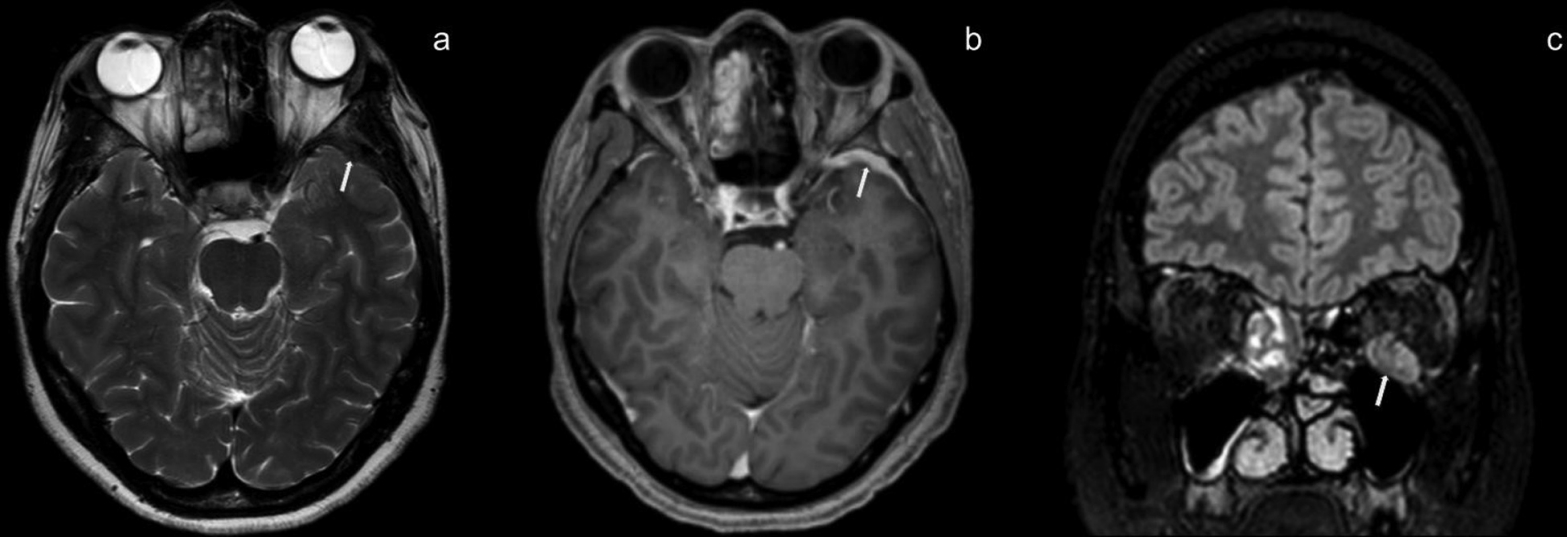
Pretreatment axial brain magnetic resonance imaging (MRI) scan showing left temporal pachymeningeal thickening adjacent to the greater sphenoid wing, with low signal intensity on T2-weighted imaging (arrow in **A** ). The corresponding postcontrast T1-weighted image demonstrates intense, homogeneous enhancement (arrow in **B** ). Coronal brain MRI scan demonstrating thickening of the left inferior rectus and inferior oblique muscles, with high signal intensity on fluid-attenuated inversion recovery (FLAIR) imaging (arrows in **C** ).
